# Simulation of diurnal variability in vertical density structure using a coupled model

**DOI:** 10.1038/s41598-021-90426-w

**Published:** 2021-05-25

**Authors:** B. Yadidya, A. D. Rao, Sachiko Mohanty

**Affiliations:** 1 Centre for Atmospheric Sciences, Indian Institute of Technology Delhi, New Delhi, 110016 India; 2 Indian Institute of Remote Sensing, ISRO, Dehradun, 248001 India

**Keywords:** Natural hazards, Ocean sciences

## Abstract

The changes in the physical properties of the ocean on a diurnal scale primarily occur in the surface mixed layer and the pycnocline. Price–Weller–Pinkel model, which modifies the surface mixed layer, and the internal wave model based on Garrett–Munk spectra that calculates the vertical displacements due to internal waves are coupled to simulate the diurnal variability in temperature and salinity, and thereby density profiles. The coupled model is used to simulate the hourly variations in density at RAMA buoy (15° N, 90° E), in the central Bay of Bengal, and at BD12 (10.5° N, 94° E), in the Andaman Sea. The simulations are validated with the in-situ observations from December 2013 to November 2014. The primary advantage of this model is that it could simulate spatial variability as well. An integrated model is also tested and validated by using the output of the 3D model to initialize the coupled model during January, April, July, and October. The 3D model can be used to initialize the coupled model at any given location within the model domain to simulate the diurnal variability of density. The simulations showed promising results which could be further used in simulating the acoustic fields and propagation losses which are crucial for Navy operations.

## Introduction

Solar heat fluxes and wind stress primarily govern the physics of the upper ocean. They affect the mixed layer in the ocean, which in turn influences the pycnocline where internal waves (IWs) are present. IWs are formed in stably stratified fluids when some external force ousts a parcel of water from its position and then restored by buoyancy forces. The restoration force depends upon the gravitational force and the density difference between the two layers in the ocean. They play a significant role in the thermodynamics of the ocean, submarine navigation, offshore oil drilling, etc. On a diurnal scale, the variations in the vertical density profiles are mainly observed in the mixed layer and the pycnocline.

The Bay of Bengal and the Andaman Sea are quite distinct from other water bodies present in the tropical region. They experience seasonally reversing monsoon winds along with massive freshwater influx from their northern boundaries. In the Indian Ocean, Kumar and Balasubramanian^1^ implemented IWAVE model based on Garrett–Munk modal spectrum. They simulated the diurnal variations in temperature and salinity for two days off the west coast of India. Sridevi et al.^2,3^ used a similar model to study the impact of IWs on sound propagation off Bhimilpatnam and Paradeep. Later, Kumar et al.^4^ coupled the Price–Weller–Pinkel^5^ (PWP) model and the internal wave (IW) model based on Garrett–Munk spectra^6^. They found that coupling these two one-dimensional models improved the simulation of vertical temperature profiles. The simulation of variations in temperature and salinity on a short time-scale is of extreme importance for naval operations as it could help them in understanding the surrounding acoustic field. However, one disadvantage from the earlier studies is that the IW model could simulate the variations only on the temporal scale. In this study, we use a different technique for solving the IW boundary value problem, which helps in simulating the displacements due to IWs on both temporal and spatial scales.

The aim of this paper is to simulate the diurnal variability of density profiles in two scenarios. In the first case, a coupled model comprising of the PWP and IW model is used when in-situ observations of temperature and salinity are available. The second case uses an integrated model, consisting of 3D MITgcm and coupled model when in-situ observations are not available. We test both model set-ups in the central Bay of Bengal and the Andaman Sea. The maximum variations in the vertical profiles of density on a diurnal scale are mainly seen in the pycnocline region. They are caused by the presence of IWs. The coupling of large-amplitude IWs with free surface waves could drastically increase the risk during strom surges and high tides along the coastal regions^7^.

## Data and methodology

The Research Moored Array for African-Asian-Australian Monsoon Analysis and Prediction (RAMA) is a tropical buoy array in the Indian Ocean^8^. RAMA buoy, located at (15° N, 90° E) is used in this study. The surface heat, momentum, and salt fluxes derived from meteorological variables are available at hourly intervals. Hydrographic data of temperature is available at a frequency of 10 minutes, and salinity is available at a one-hour frequency. Depth-wise in-situ temperature and salinity are available up to 140 m. Hourly observations of in-situ temperature and salinity up to 200 m depth at BD12 (10.5° N, 94° E), collected by the National Institute of Ocean Technology, Chennai, are also used in this study. Venkatesan et al.^9^ describe the sensors used for these observations collected in the Andaman Sea. Since the observations of meteorological variables at BD12 are not available, the surface heat, momentum, and salt fluxes are obtained from ERA5^10^ reanalysis data.

### MITgcm

**Figure 1:**
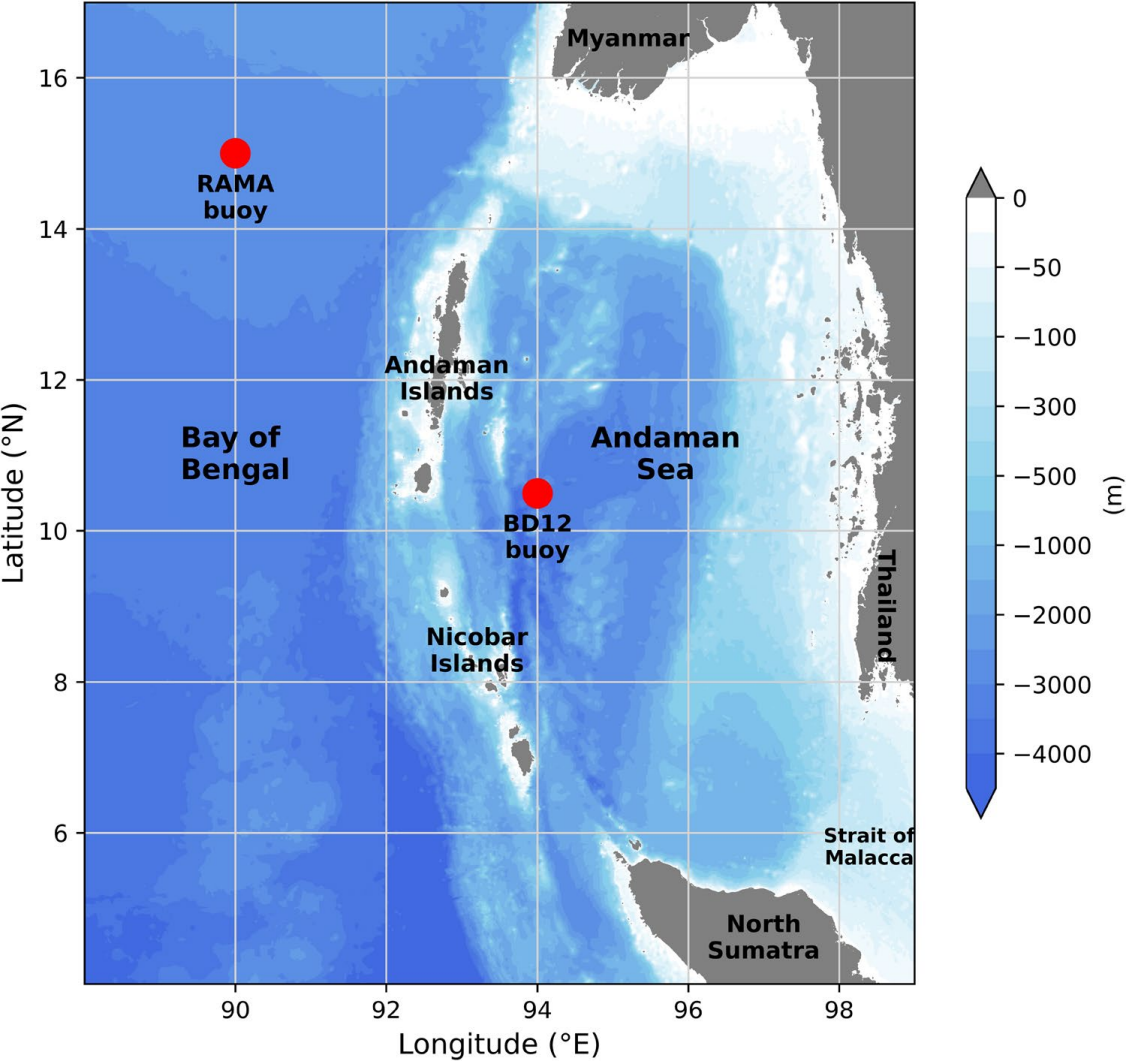
GEBCO bathymetry of MITgcm model domain along with the locations of RAMA buoy and BD12. The relevant copyright information can be found at https://www.gebco.net/data_and_products/gridded_bathymetry_data/gebco_2019/grid_terms_of_use.html.

The MITgcm model^11^ is a hydrostatic/non-hydrostatic, z-coordinate finite volume model that solves the incompressible Navier-Stokes equations with Boussinesq approximation on an Arakawa-C grid. The model domain extends from 4° N to 17° N in the meridional and 88° E to 99° E in the zonal direction with a grid resolution of 2.7 km. The bathymetry is derived from the General Bathymetric Chart of the Oceans (GEBCO)^12^. The model domain is shown in Fig. 1. There are 48 levels in the vertical. No-slip and free slip are applied at the bottom and lateral boundaries, respectively. The horizontal and vertical eddy viscosity and diffusivity are parameterized by using the Smagorinsky formulation and K-profile parameterization scheme, respectively. The value of the bottom drag coefficient is kept constant at 0.0025 in this configuration. The 3D model set-up is similar to Mohanty et al.^13^, which is used to study the energetics of internal tides in the Andaman Sea, except that the western boundary is extended to include RAMA buoy location. The same model is also used in several studies^14–16^ for understanding the IWs in the western Bay of Bengal. The model is initialized by using the temperature and salinity from Ocean Reanalysis System 5 (ORAS5). All the open boundaries of the domain are forced with the barotropic velocity components of tidal constituents (M$_2$, S$_2$, K$_1$, O$_1$) extracted from the TOPEX/Poseidon global tidal model^17^ (TPXO8). At the surface, the model is forced with daily ERA5 surface heat, momentum, and salt fluxes which have a spatial resolution of 0.25$^{\circ } \times$0.25$^{\circ }$.

### Coupled model

The PWP model uses surface heat and momentum fluxes along with the initial temperature and salinity profiles to simulate the vertical structure and evolution of the mixed layer in the ocean. It simulates the temperature and salinity of the mixed layer using three stability conditions: static stability, mixed layer stability (bulk Richardson number $\le$ 0.65), and shear flow stability (gradient Richardson number $\le$ 0.25). The PWP model accounts for free convection in the mixed layer due to surface heat loss, entrainment in the mixed layer, and mixing due to shear flow instability. The modes of IWs satisfy the eigenvalue problem

1 $$\begin{aligned} \frac{d^2 W_j(z)}{dz^2}+\{\gamma _j^2[N^2(z)-\omega _I^2]-k^2\}W_j(z)=0 \end{aligned}$$

where $W_j(z)$ are the IW modes, $\omega _I$ is the inertial frequency, $k$ is a known spatial wavenumber, $W_j(0)=W_j(H)=0$ are the boundary conditions, $H$ is the depth of water column, $\gamma _j^2$ are the eigen values related to eigen frequencies by $\omega _j^2=\omega _I^2+k^2/\gamma _j^2$, $N^2$ is the buoyancy frequency, defined as

2 $$\begin{aligned} N^2 = \frac{-g}{\rho _0}\frac{\partial \rho }{\partial z} \end{aligned}$$

where $g$ is the gravitational acceleration, $\rho _0$ is a reference ocean water density. The internal wave modes are normalized so that $\int _0^H [N^2(z)-\omega _I^2]W_j(z)W_{j^`}dz = \delta _{j,j^{'}}$. The weight function $N^2(z) - \omega _I^2$ that multiplies the eigenvalue in Eq. (1), is assumed to be positive. An alternative formulation used in the earlier studies^1–4^ treats the temporal frequency $\omega$ as known entity and attempts to find a discrete set of spatial wave numbers *k* as eigenvalues. It has a weight function that can be both negative and positive, which is not suitable for Sturm sequence method used in our study.

The finite-difference approximation is applied to Eq. (1). Thereafter, the Sturm sequence^18^ and bisection methods^19^ are used to find the eigenvalues. The inverse iteration method is used to find the eigenvectors, i.e., IW modes. Garrett and Munk have formulated an empirical model of the IW spectrum based on experimental observations. They have assumed that the IW energy is distributed over modes and frequencies that are independent of each other. The statistics provided by Garrett–Munk power spectrum along with the IW eigenvalues and modes computed from ‘finite-difference” Sturm sequence-bisection-inverse iteration’ methods are used in generating the IW displacements. A comprehensive discussion of these methods is given by Wilkinson^18^ and Evans^20^.

The PWP model and IW model based on Garrett–Munk spectra are coupled together offline to simulate the diurnal variations in density. The variations in the mixed layer of temperature and salinity are simulated by the PWP model, and then the displacements due to IWs simulated by the IW model are imposed on the mixed layer modified profiles using

3 $$\begin{aligned} T(r,z,t)= & {} T_p(z) + \zeta (r,z,t)\frac{\partial T_p}{\partial z} \end{aligned}$$

4 $$\begin{aligned} S(r,z,t)= & {} S_p(z) + \zeta (r,z,t)\frac{\partial S_p}{\partial z} \end{aligned}$$

where $T_p$, $S_p$ are temperature and salinity profiles simulated by the PWP model, $\zeta$ is the IW displacements simulated by the IW model, and $T$, $S$ are the final temperature and salinity simulated by the coupled model. The PWP model is a 1D model, whereas the IW model is 2D. Here, we assume that within a short range of about 10 km, the mixed layer does not vary significantly. Therefore, at any given time step, the gradient of temperature and salinity profiles from PWP model at the buoy location is multiplied with the displacements from the IW model within a given range (Eqs. 3 and 4) to get the IW induced fluctuations^21^. The density $\rho$ is computed using UNESCO 1983 (EOS 80) polynomial:

5 $$\begin{aligned} \rho (S,T,p)=\rho (S,T,0)/[1-\rho /K(S,T,p)] \end{aligned}$$

where $K$ is the secant bulk modulus.

The isopycnal displacement, which gives the amplitude of IWs, is computed to show their presence within a range of 10 km. It is defined as

6 $$\begin{aligned} \eta (r,z) = \frac{\rho ^{'}(r,z)}{d \overline{\rho }/d z} \end{aligned}$$

where $\overline{\rho }$ is range-mean density and $\rho ^{'}(r,z)$ is the density anomaly given by $\rho ^{'}(r,z) = \rho (r,z) - \overline{\rho }(z)$

### Experimental design

In the first model set-up, the PWP model and IW model are initialized and run with RAMA buoy observations of temperature and salinity for 24 hours starting at 00:30 hours IST. The IW model’s internal wave displacements are imposed on the mixed layer modified profiles simulated from the PWP model to get the final temperature and salinity profiles to compute the density. The same procedure is repeated every day from 1 December 2013 to 30 November 2014. The vertical resolution of the coupled model is set to 1 m by interpolating the observed profiles. The time increment and range increment are set to 1 hour and 0.5 km, respectively. The longwave and shortwave extinction coefficients are set to 0.6 m and 20 m, respectively. The first 20 modes are considered in simulating the internal wave displacements. The surface forcing is provided from observed heat, momentum, and salt fluxes. The same experiment is also conducted at BD12 but since the observations of surface fluxes are not available, ERA5 fluxes are used to force the PWP model. Henceforth, this model set-up is described as the “Coupled model”.

In the second model set-up, the 3D MITgcm is run for four different months, representing different seasons. In winter, the model is initialized on 22 December 2013 and runs until 31 January 2014. In spring, it is started on 22 March 2014 and runs until 30 April 2014. To represent the summer, the model is started on 21 June 2014 and integrated till 31 July 2014. Finally, the model is again initialized from 21 September 2014 and integrated up to 31 October 2014. The first ten days in all seasons are left out as model spin-up, and the next 30 to 31 days are used for analysis. The coupled model is initialized with the output from MITgcm at RAMA and BD12 every day at 00:30 hours in all four months and the results are analyzed. This total model set-up is mentioned as the “Integrated model”.

The first model set-up can be used when real-time observations of temperature and salinity profiles are available. They can be used to initialise the coupled model to simulate the location specific density diurnal variations. The second model set-up can be used in the absence of real-time observations by simulating the day-to-day variations using 3D MITgcm and hourly variations using coupled model. Even though the 3D MITgcm is capable of simulating hourly variations, the coupled model is preferred because of it’s simplicity and computational efficiency.

## Results and discussion

### Coupled model

**Figure 2:**
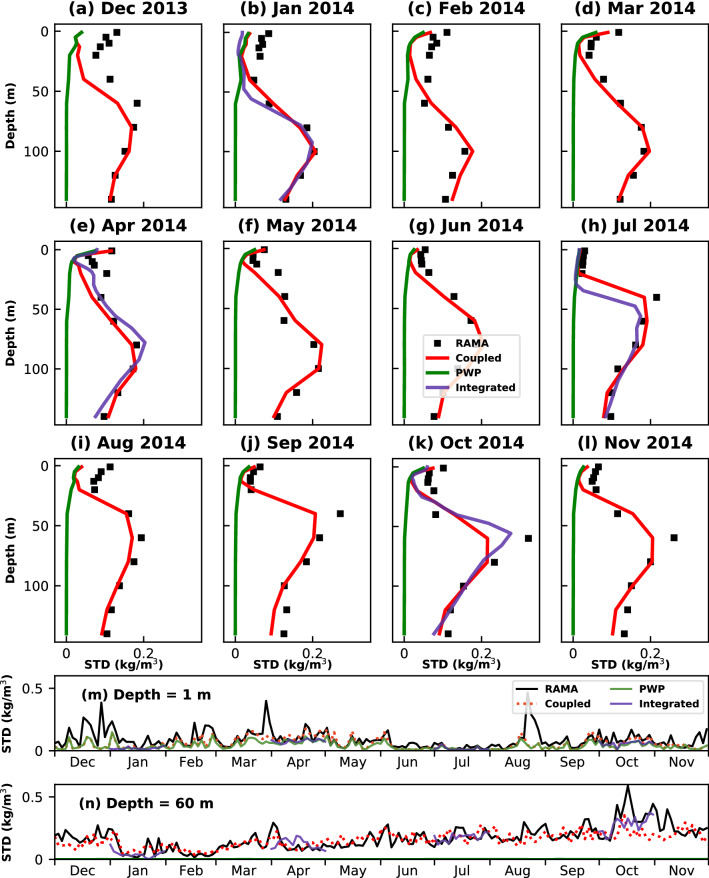
(**a**–**l**) Comparison of monthly-averaged daily standard deviation profiles of density between RAMA observations (black), Coupled model (red), PWP model (green), and Integrated model (blue). Time-series of daily standard deviation at (**m**) 1 m and (**n**) 60 m .

The daily standard deviation of density is computed from RAMA buoy observations, coupled model, and PWP model. The profiles of their monthly averaged daily standard deviation are shown and compared in Fig. 2. As the PWP model simulates the variability only within the mixed layer region, the standard deviation below the mixed layer depth is zero in all the months. During winter, a large latent heat flux is observed in the Bay of Bengal due to cool, dry, and continental air brought by northeasterly winds along with periods of high precipitation that leads to temperature inversions^22^. The daily standard deviation is very high within the upper 20 m during winter (Fig. 2a–c), especially during December (Fig. 2a). The coupled model underestimates this diurnal variability in the mixed layer. The maximum diurnal variability in January (Fig. 2b) and February (Fig. 2c) is observed at 100 m, which is captured by the coupled model. In the spring season, weak winds along with high incoming solar radiation lead to a shallow mixed layer and very high sea surface temperatures. Seasonal pycnocline (thermocline) is a common feature during this period which can be observed in the daily standard deviation profiles in April (Fig. 2e) and May (Fig. 2f). The coupled model captures the maximum diurnal variability, which is between 80 m - 100 m, during spring (Fig. 2d–f) but underestimates the variability in seasonal pycnocline. The boreal summer is the period of summer monsoon in the Bay of Bengal when it experiences very strong winds and high precipitation. The strong winds and freshwater due to precipitation deepen the mixed layer depth and decrease the diurnal variability within the mixed layer, which is clearly seen during July (Fig. 2h) when the lowest diurnal variability is observed within the upper 20 m. The maximum diurnal variability in June (Fig. 2g) is observed at 60 m, whereas in July (Fig. 2h) and August (Fig. 2i), it is seen at 40 m and 60 m, respectively, which is well captured by the coupled model. During autumn, the Bay of Bengal experiences a large discharge of freshwater flux near the head Bay of Bengal, which considerably decreases the salinity in the near-surface layers. We observed the sharpest gradients in the density diurnal variability profiles in September (Fig. 2j), October (Fig. 2k), and November (Fig. 2l) between 20 and 60 m depth. The coupled model underestimates the maximum diurnal variability of density even though it is able to simulate the trend in the profile. The time-series of daily density standard deviation near the surface at 1 m and in the pycnocline at 60 m is shown in Fig. 2m, ﻿n, respectively. Even though the coupled model underestimates the variability when the diurnal variations are very high near the surface, it is able to match the trend followed by observations.

**Figure 3:**
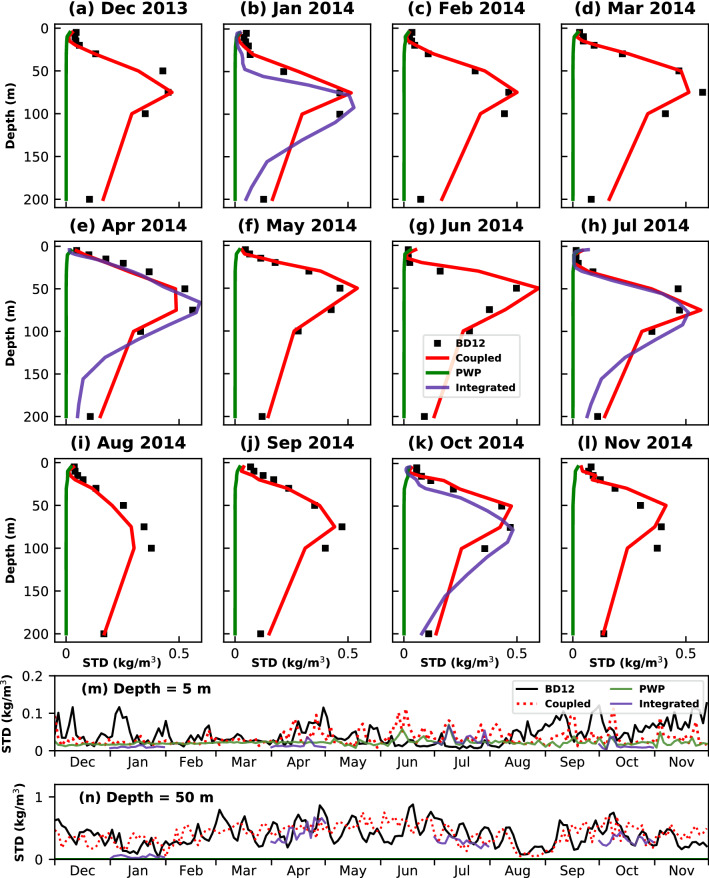
(**a**–**l**) Comparison of monthly-averaged daily standard deviation profiles of density between BD12 observations (black), Coupled model (red), PWP model (green), and Integrated model (blue). Time-series of daily standard deviation at (**m**) 5 m and (**n**) 50 m.

A similar comparison is made in the Andaman Sea with the in-situ observations available at BD12 buoy and shown in Fig. 3. The Andaman Sea is known for large-amplitude internal waves; therefore, the monthly average daily standard deviation values in the pycnocline reached 0.5 kg/m$^3$ during spring (Fig. 3g). The diurnal variability within the upper 25 m is significantly less during winter (Fig. 3a–c), with the maximum at 75 m depth. The coupled model simulates the diurnal variability reasonably well throughout the water column except at 100 m. During spring (Fig. 3d–f), the diurnal variability increased progressively from the surface and reached a maximum at 75 m before decreasing again below that. The coupled model does a good job in simulating this variability at all depths. In summer (Fig. 3g–i), each month displayed a different type of diurnal variability profiles. The maximum diurnal variability is seen at 50 m, 75 m, and 100 m in June (Fig. 3g), July (Fig. 3h), and August (Fig. 3i), respectively. The coupled model is able to capture this variability reasonably well. The daily standard deviation profiles in autumn (Fig. 3j–l) are similar to those in spring (Fig. 3d–f), with the variability increasing progressively before reaching a maximum at 75 m and decreasing below that. The coupled model simulated the diurnal density variability in all seasons but underestimated the variability at 100 m when the maximum variability is at 75 m or below that depth. This could be due to the lack of any in-situ observations between 100 m and 200 m at BD12 and having used interpolated values for model initialization. A similar trend is observed at RAMA buoy as well. Whenever the density gradient between consecutive depths is very high and interpolated values are used for model initialization, the diurnal variability is underestimated. This is clearly the case during autumn (Fig. 2j–l) at RAMA buoy. The daily standard deviation of density time-series at 5 m (Fig. 3m) and 50 m (Fig. 3n) shows that coupled model is capturing the diurnal variations throughout the year.

**Figure 4:**
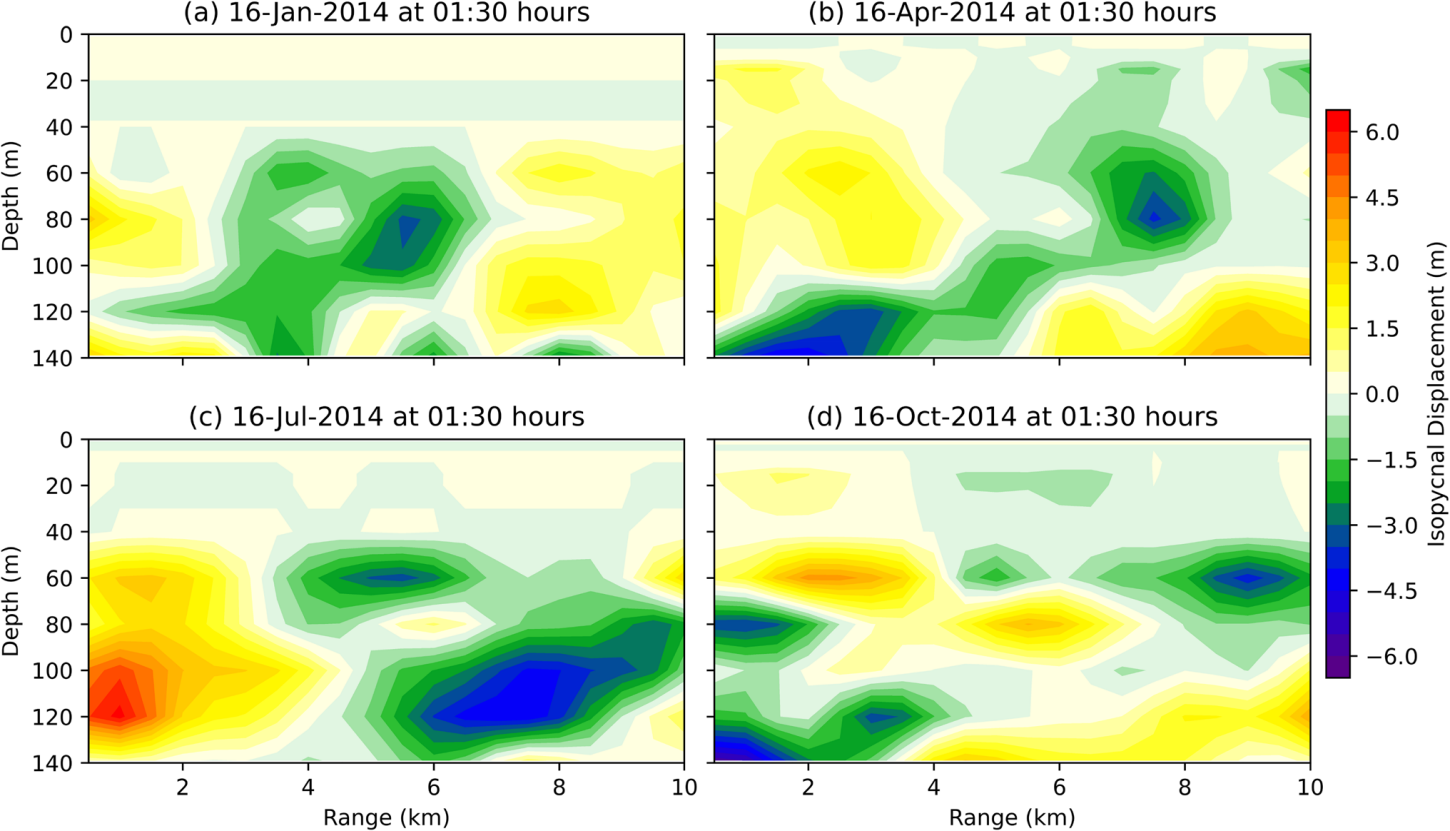
Vertical distribution of isopycnal displacements within a range of 10 km from RAMA buoy simulated by coupled model at 01:30 hours on (**a**) 16 January 2014, (**b**) 16 April 2014, (**c**) 16 July 2014, and (**d**) 16 October 2014.

The vertical distribution of isopycnal displacement simulated by the coupled model at RAMA within a range of 10 km at 01:30 hours IST is shown in Fig. 4. We can clearly observe the variation in IW amplitude within 10 km range in all seasons. The maximum isopycnal displacement of 4 m is seen at 80 m depth on 16 Jan 2014 (Fig. 4a). Whereas on 16 April 2014 (Fig. 4b) and 16 October 2014 (Fig. 4d), the maximum displacement of 4.5 m and 6.5 m, respectively, is observed at 140 m. A maximum displacement of 6.5 m is found at 120 m on 16 July 2014 (Fig. 4c). The presence of these strong displacements within a 10 km range in the pycnocline indicate the presence of IWs and this information is crucial for Navy operations. The temperature and salinity profiles used to compute the density can be further used to simulate and study the acoustic field along with the acoustic transmission losses.

### Integrated model

The integrated model, at both RAMA (Fig. 2b,e,h,k) and BD12 (Fig. 3b,e,h,k), is able to simulate the diurnal variability in density observations reasonably well except near the base of the mixed layer. This is seen during January 2014 (Figs. 2b and 3b) at both RAMA and BD12 and in July (Fig. 2h) at RAMA, where the integrated model underestimates the variability just below the mixed layer. This could be due to bias in the initial conditions provided to the MITgcm from ORAS5^23^, especially in the salinity profiles, which mainly control the stratification in the upper layers of the water column in the Bay of Bengal and the Andaman Sea. However, even with this limitation, the integrated model does a better job than the coupled model in two exceptional cases. One, it is able to capture the variability within the seasonal pycnocline better than the coupled model in April (Fig. 2e) at RAMA. Secondly, the integrated model does a better job in capturing the maximum diurnal variability in October (Fig. 2k) at RAMA, where the density gradients are very high at 60 m. The integrated model also reduces the error in estimating the diurnal variability near 100 m at BD12 in January (Fig. 3b) and October (Fig. 3k). This could be primarily attributed to better vertical spatial resolution in the integrated model. For example, the observations at RAMA are available at 40 m, 60 m, and 80 m. Whereas in the integrated model set-up, the initial profiles of temperature and salinity are available at every 5 m interval between 40 and 80 m.

## Conclusions

The PWP model is coupled with the IW model based on Garrett–Munk spectra to simulate the diurnal and spatial variability in the density profiles in the central Bay of Bengal and the Andaman Sea. The IW eigenvalue problem is solved with ‘finite-difference - Sturm sequence - bisection - inverse iteration’ method which helps in simulating the spatial distribution of IW displacements along with the temporal variability. This is a major improvement from earlier studies^1–4^, where they could not simulate the spatial variability within a given range. The coupled model simulations are validated by using the daily standard deviation profiles for different months at RAMA and BD12. The diurnal variability of density showed significant monthly and seasonal variations in both central Bay of Bengal and Andaman Sea and the coupled model is able to simulate the variability reasonably well. One limitation of this model is that it underestimates the diurnal variability when interpolated values are used for model initialization in the presence of a high density gradient.

We also used an integrated model by initializing the coupled model with output from 3D MITgcm for four different months representing different seasons. The integrated model does a better job in simulating the diurnal variability in the presence of seasonal pycnocline and sharp density gradients in the main pycnocline. This is due to the higher vertical resolution in the initialization profiles taken from 3D MITgcm. However, the integrated model underestimates the variability at the base of the mixed layer. This could be attributed to poor representation of salinity profiles in ORAS5 which are used to intialize the 3D model.

In summary, simulations from two model set-ups - coupled model and integrated model, are validated in the Bay of Bengal and the Andaman Sea in different months, and the error is statistically quantified. These models could be further used to study the acoustic field and propagation losses temporally as well as spatially. The concept of using the integrated model for simulating the diurnal variability of the ocean environment can become really handy for Navy operations. Since taking observations of temperature and salinity in real-time could be difficult, the integrated model can be used to simulate the temporal and spatial variations with a reasonable degree of accuracy to further understand the acoustic fields. Furthermore, the improvement in the initial salinity fields provided to the integrated model could help reduce the error in the simulations.

## Data Availability

The altimeter data is from Global Ocean Gridded L4 Sea Surface Heights and Derived Variables Reprocessed - Metadata provided by E.U. Copernicus Marine Service Information. The RAMA buoy data can be downloaded from https://www.pmel.noaa.gov/tao/drupal/disdel/. The BD12 buoy data is available upon request.
